# YKL-40 Aggravates Early-Stage Atherosclerosis by Inhibiting Macrophage Apoptosis in an Aven-dependent Way

**DOI:** 10.3389/fcell.2021.752773

**Published:** 2021-12-07

**Authors:** Wei Huan, Liu Yandong, Wang Chao, Zou Sili, Bai Jun, Liao Mingfang, Chen Yu, Qu Lefeng

**Affiliations:** ^1^ Department of Vascular and Endovascular Surgery, Second Affiliated Hospital of Naval Medical University, Shanghai, China; ^2^ Yueyang Hospital of Integrated Traditional Chinese Medicine & Clinical Research Institute of Integrative Medicine, Shanghai University of Traditional Chinese Medicine, Shanghai, China

**Keywords:** atherosclerosis, macrophage, apoptosis, YKL-40, aven

## Abstract

**Objective:** programmed cell removal in atherosclerotic plaques plays a crucial role in retarding lesion progression. Macrophage apoptosis has a critical role in PrCR, especially in early-stage lesions. YKL-40 has been shown to be elevated as lesions develop and is closely related to macrophages. This study aimed to determine the effect of YKL-40 on regulating macrophage apoptosis and early-stage atherosclerosis progression.

**Research design and Methods:** The correlations among the expression level of YKL-40, the area of early-stage plaque, and the macrophage apoptosis rate in plaques have been shown in human carotid atherosclerotic plaques through pathological and molecular biological detection. These results were successively confirmed *in vivo* (*Ldlr* ^−/-^ mice treated by YKL-40 recombinant protein/neutralizing antibody) and *in vitro* (macrophages that *Ykl40* up-/down-expressed) experiments. The downstream targets were predicted by iTRAQ analysis.

**Results:** In early-stage human carotid plaques and murine plaques, the YKL-40 expression level had a significant positive correlation with the area of the lesion and a significant negative correlation with the macrophage apoptosis rate. *In vivo*, the plaque area of aortic roots was significantly larger in the recomb-YKL-40 group than that in IgG group (*p* = 0.0247) and was significantly smaller in the anti-YKL-40 group than in the IgG group (*p* = 0.0067); the macrophage apoptosis rate of the plaque in aortic roots was significantly lower in the recomb-YKL-40 group than that in IgG group (*p* = 0.0018) and was higher in anti-YKL-40 group than that in VC group. *In vitro*, the activation level of caspase-9 was significantly lower in RAW264.7 with *Ykl40* overexpressed than that in controls (*p* = 0.0054), while the expression level of Aven was significantly higher than that in controls (*p* = 0.0031). The apoptosis rate of RAW264.7 treated by recomb-YKL40 was significantly higher in the *Aven* down-regulated group than that in the control group (*p* < 0.001). The apoptosis inhibitor Aven was confirmed as the target molecule of YKL-40. Mechanistically, YKL-40 could inhibit macrophage apoptosis by upregulating Aven to suppress the activation of caspase-9.

**Conclusion:** YKL-40 inhibits macrophage apoptosis by upregulating the apoptosis inhibitor Aven to suppress the activation of caspase-9, which may impede normal PrCR and promote substantial accumulation in early-stage plaques, thereby leading to the progression of atherosclerosis.

## Introduction

Cardio- and cerebrovascular diseases are the leading causes of mortality worldwide and account for well over 20% of all deaths, according to World Health Organization (WHO) estimates (Mathers and Loncar, 2006; Kim and Johnston, 2011). Statistics show that 9–35% of transient ischemic attacks (TIA) or strokes are associated with extracranial carotid atherosclerosis (Sacco et al., 1995; Wityk et al., 1996). Currently, treatments for carotid artery stenosis are most often performed at the late stage of carotid atherosclerosis, aiming to improve cerebral ischemia symptoms and prevent stroke or other serious ischemic complications. There are no definite effective means to inhibit plaque aggravation in the early stage to date.

Atherosclerosis is universally identified as a progressive inflammatory disease. In its initial stage, apolipoprotein B-containing lipoproteins accumulate in the arterial intima, triggering a chronic inflammatory response dominated by the recruitment of monocyte-derived macrophages (Tabas and Lichtman, 2017). Macrophages engulf lipids and form foam cells, a hallmark of the onset of early-stage atherosclerosis. Then apoptosis occurs and the cellular debris of macrophages is removed by efferocytosis. This programmed cell removal (PrCR) process can reduce the components of atherosclerotic plaque, which would effectively decrease plaque burden and inhibit lesion progression (Tabas, 2005; Vandivier et al., 2006; Gautier et al., 2009; Seimon and Tabas, 2009). Studies have shown that the inhibition of macrophage apoptosis significantly exacerbates the progression of early-stage atherosclerosis (Liu et al., 2005). Though the effect of macrophage apoptosis on atherosclerosis progression is reverse in advanced lesions, it is thought to be caused by impaired efferocytosis of phagocytes in the later-stage lesions (Tabas, 2005; Schrijvers et al., 2007). In our previous research, the impaired efferocytosis function of macrophages induced by the inflammatory factor YKL-40 contributed to enlarging the necrotic core and undermining the stability of later-stage atherosclerosis plaques.

YKL-40, also known as chitinase-3-like protein 1 (CHI3L1), is a 40 kD mammalian glycoprotein, belonging to the conserved mammalian chitinase family but without chitinase activity (Hakala et al., 1993; Rathcke and Vestergaard, 2006). YKL-40 has been identified as a novel biomarker of systemic inflammation and is expressed and secreted by macrophages, vascular smooth muscle cell (VSMC) and neutrophils under pathological conditions (Michelsen et al., 2010). Recent studies have shown that YKL-40 could play an important role in regulating apoptosis, pyroptosis, and activation of inflammasomes, etc. (Lee et al., 2009; Sohn et al., 2010; Chen et al., 2011; Areshkov et al., 2012). YKL-40 is lack of chitinase activity, while it still reserves certain biological function like possessing the carbohydrate-binding motif (CBM), which is considered the potential path that YKL-40 enhancing the AKT phosphorylation (Chen et al., 2011). Our previous research and other studies have found that serum levels of YKL-40 are markedly increased in patients with carotid atherosclerosis, especially in patients with ischemic symptoms (Kjaergaard et al., 2010; Michelsen et al., 2010; Wu et al., 2013). Moreover, a large population study confirmed that elevated plasma YKL-40 levels were positively correlated with an increased risk of ischemic stroke (Kjaergaard et al., 2010). We have known that YKL-40 could aggravate atherosclerosis progression. However, the effect of YKL-40 on the function of macrophages and the PrCR process in plaque and its mechanisms during early-stage atherosclerosis are still not known.

In this study, we focused on the effect of YKL-40 on regulating early-stage atherosclerotic plaque progression and explored its potential mechanisms.

## Materials and Methods

### Human Study Population and Sample Preparation

Early-stage carotid plaques and corresponding blood samples were collected from 16 patients with carotid body tumors and five patients with carotid aneurysm undergoing surgical operations in the Department of Vascular and Endovascular Surgery, Changzheng Hospital, affiliated with the Naval Medical University (Shanghai, People’s Republic of China) between July 2015 and September 2019. The inclusion criteria were patients with inchoate atherosclerosis in the carotid arteries. Exclusion criteria were patients with definite atherosclerotic carotid stenosis and renal/liver failure or other chronic disease that might result in metabolic abnormalities. As previously described, early-stage plaque samples were collected including foam cells and lipid particles accumulating in several pools in the thickening intima. Advanced lesions that had developed a massive, confluent, well-delineated accumulation of extracellular lipid (a lipid core) were excluded (Stary et al., 1994). For this study, all plaque samples were collected from patients intraoperatively, then flushed with phosphate buffered saline (PBS) and immediately stored in liquid nitrogen. All peripheral blood samples were collected before the procedure. Serum was acquired by centrifugation of the peripheral blood at 3,500 rpm for 10 min at room temperature and stored at −80°C before further processing. This research was conducted according to institutional and ethical guidelines. Patients’ informed consents were obtained. The study protocol was approved by the Independent Ethics Committee of the Changzheng Hospital affiliated with the Naval Medical University.

### Calculating Relative Area and Grouping for Samples

H&E (hematoxylin-eosin) staining was performed on the cross-section of the plaques. The plaque area and total lesion area were calculated according to the staining outcomes, and the percentage of plaque area to total lesion area was counted as the relative area (%) of plaque (Supplementary Figure S1).

In order to compare the results between the groups on the basis of lesion size, all 21 samples were divided into a small group (*n* = 10) and a large group (*n* = 11) at the median value (51.32%) of plaque relative area. Baseline information on these patients in the two groups are compared in Supplementary Table S1.

### Atherosclerosis Animal Model and *In Vivo* Administration Protocol

*Ldlr* ^–/–^ mice were bred based on C57BL/6 mice obtained from Jackson Laboratory. Male *Ldlr* ^–/–^ mice were weaned at 3 weeks old and fed a chow diet until 6 weeks old, then a high cholesterol diet (HCD) was maintained (0.4% fat +1.1% cholesterol +18.8% casein, Research Diets, America, D12104C) to construct the atherosclerosis model. The atherosclerotic plaque collected from the aorta of *Ldlr* ^*–/–*^ mice fed a HCD ≤12 weeks were regarded as early-stage lesions, according to the histological morphology and the criterion as previously described (Whitman, 2004).

At 12 weeks old (while HCD feeding has been lasted for 6 weeks), the administration was initiated. *Ldlr* ^*–/–*^ mice were randomly divided into three groups (*n* = 5), including the control group (IgG), YKL-40 recombinant protein group (recomb-YKL-40), and YKL-40 neutralizing antibody group (anti-YKL-40). At each week of age, IgG (1 mg/g), YKL-40 recombinant protein (500 ng/g) and YKL-40 neutralizing antibody (1 mg/g) was intraperitoneally injected for six successive weeks, while the high cholesterol diet was continued. Mice were euthanized after administration completed (while HCD feeding has been lasted for 12 weeks) and tissues were collected. Customized YKL-40 neutralizing antibody (Huiou Co., Ltd., Shanghai, China) was extracted from rabbit serum according to previously described (Mizoguchi, 2006) and the titer of the antiserum reached to 1:72,900. The administration program was determined by the relatively large dose of agents (1 mg/g) and the protocols used in the previous studies (Kojima et al., 2016). After mice were euthanized, cold PBS was injected through the left ventricle to flush out blood or thrombus followed by an injection of 4% paraformaldehyde (10 ml per mouse) for internal fixation. The murine aorta tissue (from aortic root to iliac artery) was removed, the aortic root was immediately stored in liquid nitrogen, and the residual part was fixed in 4% formaldehyde at 4°C for further assay.

### Histology and Immunofluorescent Staining

The human carotid plaques and murine aortic root were embedded in paraffin or optimal cutting temperature compound, followed by cutting into 5-μm-thick cryosections. H&E and Masson trichome staining was performed to examine plaque morphology. Human early-stage plaques were defined as previously described (Stary et al., 1994), according to the morphological assay. The murine aortic root cryosections and residual aorta tissue were stained with oil-red O for determination of lipids. Immunofluorescence staining was performed as previously described (Kojima et al., 2016). Briefly, after being blocked in 1% bovine serum albumin (BSA), sections were incubated with primary antibodies: human macrophages labeled with anti-CD68 Ab (Abcam ab201340 1:100); mouse macrophages labeled with anti-MOMA-2 Ab (Abcam ab33451 1:50); and human/mouse YKL-40 labeled with anti-YKL-40 Ab (Abcam ab180569 1:100). After being washed with PBS, the sections were incubated with fluorescein isothiocyanate (FITC)-conjugated secondary antibodies. Nuclei were stained with 4′, 6-diamidino-2-phenylindole (DAPI; 1:2000) for 5 min. After being rinsed three times in PBS, the sections were examined under a fluorescence microscope. The immunostaining was measured by ImageJ software.

### Biochemical Assay

Mice were euthanized after being fasted overnight. Murine blood samples were centrifuged for collecting serum. Total cholesterol (TC), triglycerides (TG), low-density lipoprotein (LDL), and high-density lipoprotein (HDL) were measured by enzymatic colorimetric assays using colorimetric kits (Jiancheng, Nanjing, China) with an automatic biochemical analyzer.

### Enzyme-Linked Immunosorbent Assay

Serum YKL-40 levels were measured with the related ELISA kits (R&D DC3L10) according to the manufacturer’s instructions.

### Measurement of Macrophage Apoptosis Rate Detection in Human and Murine Plaques

Human carotid plaque and murine aortic root sections were double stained for macrophages and apoptotic cells. Macrophages were labeled with anti-CD68 Ab (Abcam ab201340 1:100) or anti-MOMA-2 Ab (Abcam ab33451 1:50) antibody and apoptotic cells were stained with TUNEL kits (Progema G3250) according to the manufacturer’s instructions. The proportion of TUNEL^+^ in CD68^+^ cells in each sample was calculated as the apoptosis rate of macrophages in the plaque. Nine randomized magnified fields were chosen to measure the apoptosis rate.

### Cell Culture

Mouse bone marrow-derived macrophage (BMDM) were isolated from male C57BL/6 mice and differentiated in Dulbecco’s modified Eagle’s medium (DMEM) supplemented with 10% fetal bovine serum, 1% penicillin/streptomycin (Invitrogen), and 10 ng/ml murine M-CSF (Peprotech). The murine macrophage cell line RAW264.7 (ATCC, SC-6003) was grown in DMEM growth media containing 10% fetal bovine serum (FBS) and 1% penicillin/streptomycin (Invitrogen). Cells were cultured in a 5% CO2 humidified atmosphere at 37°C.

### *In Vitro* Apoptosis Rate Measurement

For the adherent cell assays, BMDM were embedded into Lab-Tek^®^ chamber slides (Sigma). Apoptotic BMDM were induced via Ox-LDL (100 μg/ml for 24 h) combined with staurosporine (STS) (1 μM for 4 h). The apoptotic cells were stained with TUNEL kits (Progema G3250) according to the instructions. Nine randomized magnified fields were chosen to measure the apoptosis rate.

For the flow cytometry-based assays, apoptosis-induced BMDM were prepared into cell suspension. Apoptotic BMDM were detected with the FITC Annexin V Apoptosis Detection Kit with PI (BioLegend 640,914), according to the manufacturer’s instructions. Apoptotic cells were quantified via flow cytometry using the FACS verse cell analyzer (Becton-Dickinson).

### Plasmid Transfection

RAW264.7 were prepared in cell suspension then plated at 1–2 × 10^5^ cells/well in a 24-well plate and cultured for 24 h. Commercially available plasmids for over-expressing or silencing *Ykl40*/*Aven* (Genechem Co.,Ltd., Shanghai, China) were used. Plasmids were transfected using Lipofectamine 3,000 (Invitrogen L3000001) according to the manufacturer’s instructions. Cells were passaged or harvested for further experiments 48–72 h after transfection.

### iTRAQ Labeling

iTRAQ-based proteomics analysis (Genechem Co.,Ltd., Shanghai, China) were performed to screen out the potential downstream target molecules of YKL-40. Three biological replicates were used for global proteome analysis. BMDM was treated with YKL-40 (500 ng/ml) or IgG as a control for 24 h to assay the potential pathways involved. We collected 20 mg protein from each sample and loaded into 12% SDS-PAGE for electrophoresis. The samples then underwent filter-aided sample preparation and were dissolved in 5X dissolution buffer. Then 100 μg peptide fragments from each sample were labeled with iTRAQ-8 plex reagents according to the protocol from AB SCIEX. The labeled peptides were separated by high-performance liquid chromatography (Agilent 1,260 Infinity II HPLC), followed by mass spectrometric analysis and identification. The raw data were processed with Mascot 2.5 software and Proteome Discoverer 2.1 to search the database. The experiment had been performed in our previous research, and the results were further explored in this study. The mass spectrometry proteomics data have been deposited to the ProteomeXchange Consortium with identifier PXD028305 via the PRIDE partner repository.

### Western Blot Assay

Cells and homogenated human plaques were lysed in RIPA lysis buffer (ThermoFisher) for 60 min at 4°C. Protein samples were subjected to 10% Bis-Tris gels, electrophoresed, transferred to Immobilon-FL membranes (Millipore), and probed with the indicated primary antibody: caspase-3 (CST 9662S), cleaved caspase-3(Asp175) (CST 9661T), caspase-9 (CST 9508T), Aven (CST 2300S), Akt (CST 9272S), Phospho-Akt (Thr308) (CST 13038T), and β-Actin (CST 4970S), followed by incubation with horseradish peroxidase-conjugated secondary antibodies. Protein bands were visualized using an LAS 3000 Imager (Fujifilm) and were quantified using ImageJ software (ImageJ 1.51U, Fuji edition).

### Ribonucleic Acid Extraction and Quantitative Real-Time Polymerase Chain Reaction

RNA was isolated from samples and cells using the RNeasy Kit (QIAGEN), and cDNA was synthesized from RNA (500 ng–1 μg per reaction) using Prime Script RT Master Mix (TaKaRa 2,621). Quantitative Real-time PCR (qRT-PCR) was performed using the LightCycler 480 Real-Time PCR System (Roche) and SYBR Green I Master (Roche 04,707,516,001). Primer sets used are listed in supplemental data (Supplementary Table S1).

### Statistics Analysis

Independent experiments were replicated at least three times. All results are represented as mean ± SEM. Normality was determined using D’Agostino-Pearson and/or Shapiro-Wilk normality testing. *p*-values for normally distributed data were calculated using Student’s *t*-test or one-way ANOVA analysis. *p*-values for non-normally distributed data were calculated using the Mann-Whitney U test. Percentages were analyzed with Chi-square testing or Fisher’s exact test. Correlation coefficients (*r*) and related *p*-values were calculated using the Pearson product-moment correlation analysis. Statistical analysis was performed using Graphpad Prism 8.1. A *p*-value of <0.05 was deemed statistically significant.

## Results

### Positive Correlation Was Determined Between Expression Level of YKL-40 and Carotid Early-Stage Plaque Area

**FIGURE 1:**
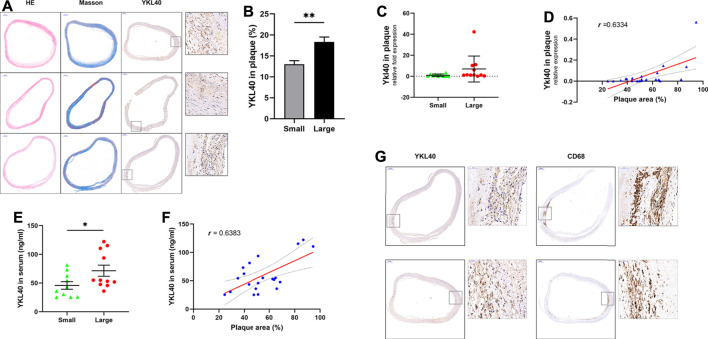
**(A)** H&E, Masson staining and anti-YKL-40 Ab labeling were performed on carotid plauqe samples. **(B)** Relative expression level of YKL-40 in unit area of plaques were calculated based on immunohistochemistry. The YKL-40 expression level in the large group was significantly higher than in the small group (*p* = 0.0034). **(C,D)** The mRNA expression level of YKL-40 in plaque tissues were detected via qRT-PCR. Significant positive correlation existed between the Ykl40 expression level in the plaque tissues and plaque relative area (*r* = 0.6334, *p* = 0.0021), but the difference between two groups was not significant (*p* > 0.05). **(E,F)** YKL-40 expression level in serum samples were detected by ELISA assay. The YKL-40 expression level in serum was significantly higher in the large group than in the small group (*p* = 0.0447) and was significantly positively correlated with plaque relative area (*r* = 0.6383, *p* = 0.0018). **(G)** Colocalization between YKL-40 and macrophages in plaque tissues as indicated by immunohistochemical detection.

The expression level of YKL-40 in the large group of human subjects was significantly higher than in the small group, according to immunohistochemical staining (*p* = 0.0034, Figures 1A,B). Significant positive correlation was confirmed between the *Ykl40* expression level in the plaque tissues and relative area of plaques (*r* = 0.6334, *p* = 0.0021, Figure 1D). Although the mean of *Ykl40* expression level was higher in the large group than in the small group, the difference was not significant (*p* > 0.05, Figure 1C), which may be due to the relatively low expression level of *Ykl40* in plaques. However, the expression level of YKL-40 in serum, which was detected by enzyme-linked immunosorbent assay (ELISA), was significantly higher in the large group than in the small group (*p* = 0.0447, Figure 1E) and was significantly positively correlated with plaque relative area (*r* = 0.6383, *p* = 0.0018, Figure 1F). These results indicated the positive correlation between the expression level of YKL-40 and the relative area of early-stage carotid plaque. Moreover, the location of YKL-40 had a significant correlation with CD68 in plaque tissues according to immunohistochemical staining (Figure 1G). The result suggests the potential association between YKL-40 and macrophages.

### Negative Correlation Was Confirmed Between the Apoptosis Rate of Macrophages in Early-Stage Carotid Plaque Samples and the Lesion Area or the Serum Level of YKL-40

**FIGURE 2:**
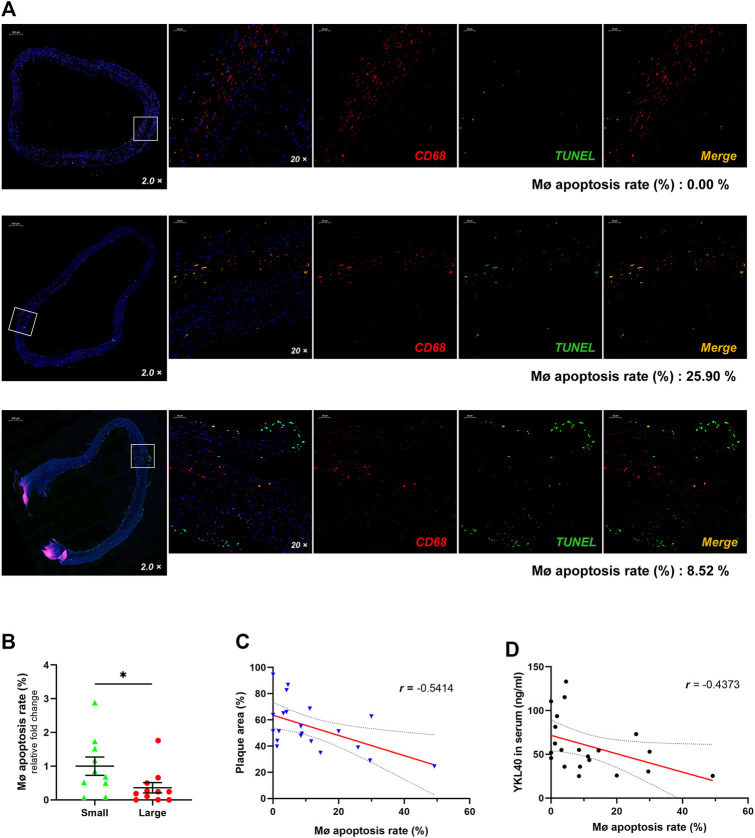
**(A)** The immunofluorescent double-labelled staining for carotid plaque samples. CD68^+^was presenting as red fluorescence while TUNEL^+^ is shown as green and Merge as golden. The proportion of TUNEL^+^ in CD68^+^ cells was calculated as the apoptosis rate of macrophages, which is shown beneath each panel. **(B)** The macrophage apoptosis rate in the large group was significantly lower than that in the small group (*p* = 0.0497). **(C)** There was a significant negative correlation between the macrophage apoptosis rate and relative plaque area (*r* = −0.5414, *p* = 0.0112). **(D)** There was a significant negative correlation showed between the macrophage apoptosis rate and expression level of YKL-40 in serum (*r* = −0.4373, *p* = 0.0475).

Immunofluorescent double-labelled staining for macrophages (CD68) and apoptotic cells (TUNEL) was performed on the above 21 human carotid plaque samples (Figure 2A). The macrophage apoptosis rate in the large group was significantly lower than that in the small group (*p* = 0.0497, Figure 2B). Moreover, a significant negative correlation was confirmed between the macrophage apoptosis rate in plaques and relative plaque area (*r* = -0.5414, *p* = 0.0112, Figure 2C). Meanwhile, a significant negative correlation was also confirmed between the macrophage apoptosis rate in plaques and expression level of YKL-40 in serum (*r* = −4,373, *p* = 0.0475, Figure 2D). These results indicated that the apoptosis of macrophages is strongly associated with YKL-40.

### No Differences in Weight or Lipid Metabolism Among *Ldlr* ^−/-^ Mice of Three Groups

**FIGURE 3:**
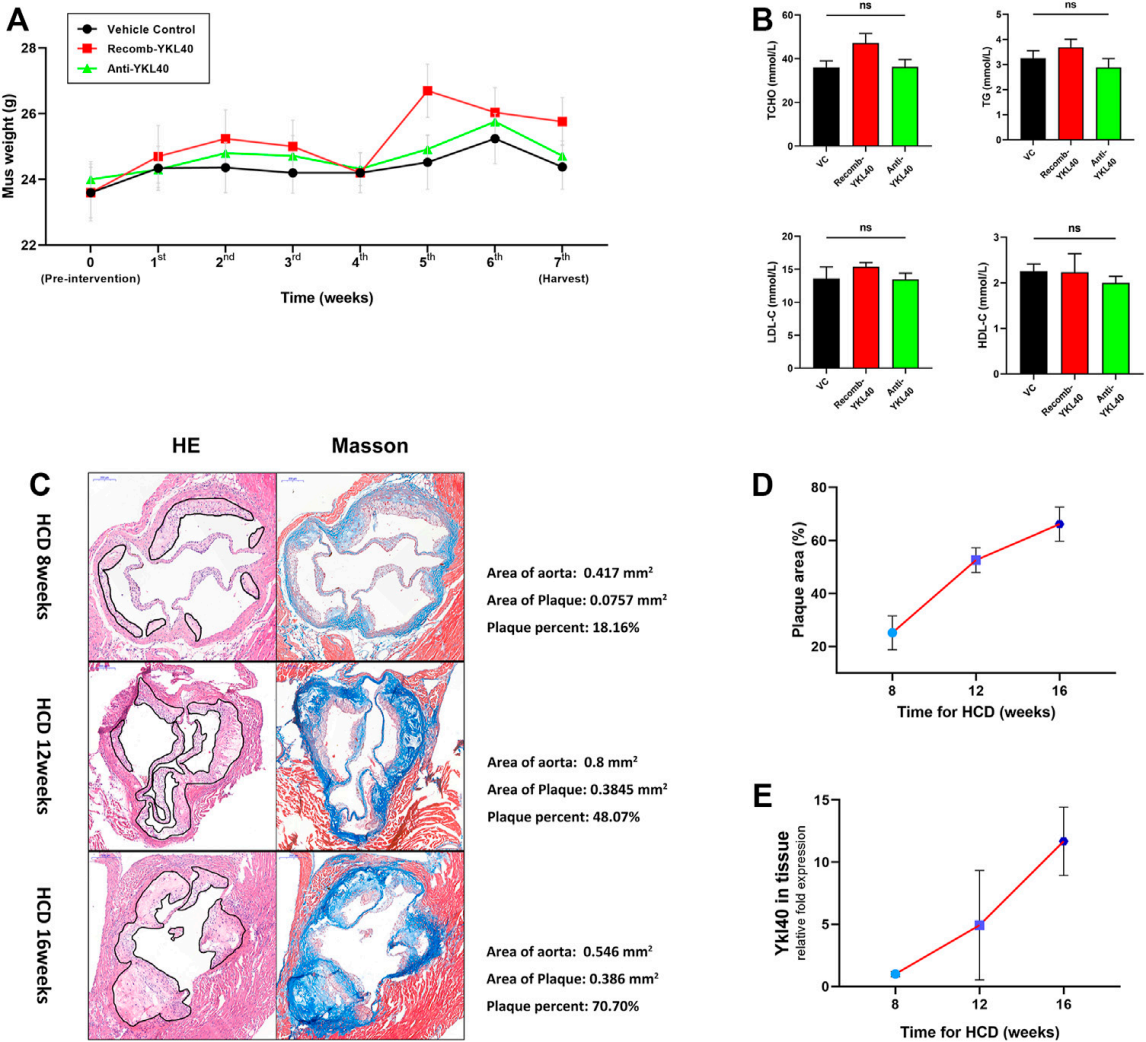
**(A)** Weight curve of mice in different groups during intervention. No statistical differences were confirmed. **(B)** Blood lipid (TCHO, TG, LDL-c, HDL-c) levels of mice in different groups. No statistical differences were confirmed. **(C)** H&E and Masson staining on aortic valves of mice at 8, 12, and 16 weeks (the area of plaque is indicted by black lines). Calculations of the relative area are shown on the right side. **(D)** Increasing trend of aortic root plaque relative area as modeling time advanced. **(E)** Increasing trend of *Ykl-l40* expression level on the aortic arch as modeling time increased.

Weights of all *Ldlr* ^−/-^ mice in IgG group, YKL-40 recombinant protein group (recomb-YKL-40), and YKL-40 neutralizing antibody group (anti-YKL-40) were monitored and recorded weekly from the last day before intervention to the last day before harvest. Sera from all these mice were collected after euthanasia to test blood lipid levels including total cholesterol (TCHO), triglycerides (TG), high-density lipoprotein cholesterol (HDL-C), and low-density lipoprotein cholesterol (LDL-C). No statistical differences in weight or blood lipid levels of mice were found among the three groups, which indicated that the nutrition status and lipid metabolism of *Ldlr* ^−/−^mice were not affected by the intervention of the YKL-40 recombinant protein or YKL-40 neutralizing antibody (*p* > 0.05, Figures 3A,B).

### Positive Correlation Was Confirmed Between the Plaque Area in Aortic Root and Expression Level of YKL-40 in *Ldlr* ^−/-^ Mice

In the process of establishing the atherosclerotic mice model by HCD feeding, we randomly selected mice at 8, 12, and 16 weeks, respectively, to obtain the tissue samples, including aortic root and aortic arch. H&E and Masson staining were performed on the aortic root to measure the relative area of plaque. We detected the expression level of *Ykl40* in aortic arch tissues by qRT-PCR. Evidently, atherosclerotic plaques were formed on aortic roots at all three time periods, which confirmed the success of model construction (Figure 3C). Meanwhile, both the relative plaque area in the aortic root and *Ykl40* expression level in the aortic arch showed a consistently increasing trend as the HCD feeding time increased. The result confirmed the positive correlation between the progression of the plaque area and expression level of YKL-40 in *Ldlr* ^−/-^ mice (Figures 3D,E).

### YKL-40 Resulted in Progression of Atherosclerotic Plaque in *Ldlr* ^−/-^ Mice

**FIGURE 4:**
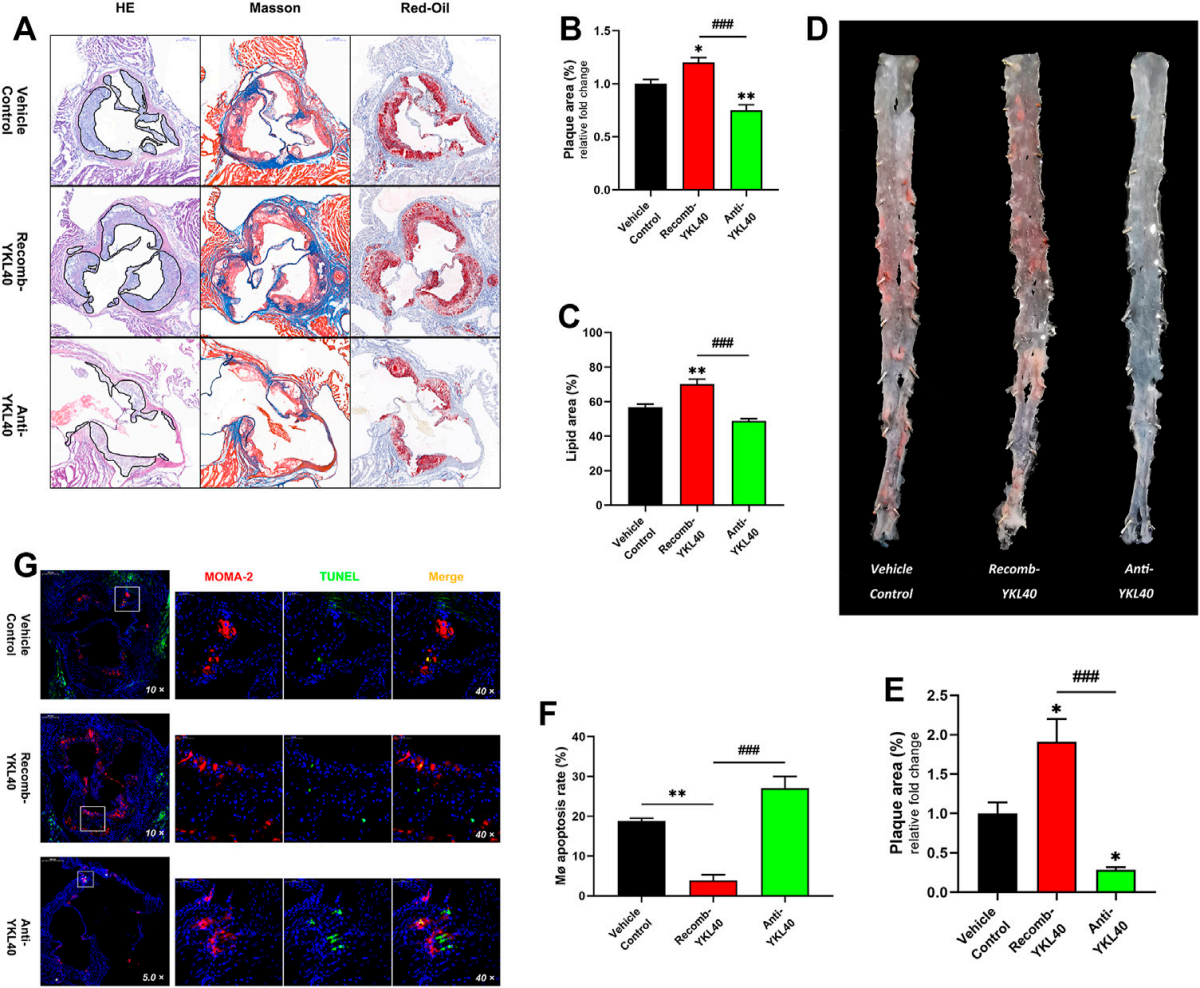
**(A–C)** H&E, Masson, and oil-Red O staining of aortic roots (the area of plaque is outline in black). The plaque area of the aortic root was significantly larger in the recomb-YKL-40 group than that in IgG group (*p* = 0.0247) and in the anti-YKL-40 group (*p* < 0.0001), and which was significantly smaller in the anti-YKL-40 group than in the IgG group (*p* = 0.0067). The lipid area was significantly larger in the recomb-YKL-40 group than that in the IgG group (*p* = 0.0028) and in the anti-YKL-40 group (*p* = 0.0001). **(D,E)** Oil-Red O staining of the whole aortas. The plaque area of the whole aorta was significantly larger in the recomb-YKL-40 group than that in IgG group (*p* = 0.0126) and in the anti-YKL-40 group (*p* = 0.0001) and was significantly smaller in the anti-YKL-40 group than in the IgG group (*p* = 0.0464). **(F,G)** Immunofluorescence and TUNEL staining were performed on aortic root plaques (red indicates MOMA-2, green for TUNEL, and golden for MERGE). The macrophage apoptosis rate in aortic root plaques was significantly lower in the recomb-YKL-40 group than that in IgG group (*p* = 0.0018) and anti-YKL-40 group (*p* = 0.001) and was higher in the anti-YKL-40 group than that in the IgG group, but the difference was not statistically significant (*p* = 0.0513).

H&E, Masson, and oil-Red O staining were performed on aortic roots, and the plaque area as well as the lipid area were measured in the three mice groups (IgG, YKL-40 protein, YKL-40 neutralizing antibody) (Figure 4A). Oil-red O staining was performed on the whole aorta to measure the plaque relative area as well (Figure 4D). The plaque area in the aortic root was significantly larger in the recomb-YKL-40 group than that in IgG group (*p* = 0.0247) and was significantly smaller in the anti-YKL-40 group than in the IgG group (*p* = 0.0067, Figure 4B). The lipid area was significantly larger in the recomb-YKL-40 group than that in the IgG group (*p* = 0.0028) and in the anti-YKL-40 group (*p* = 0.0001, Figure 4C). Similarly, the plaque area of the whole aorta was significantly larger in the recomb-YKL-40 group than that in the IgG group (*p* = 0.0126) and was significantly smaller in the anti-YKL-40 group than in the IgG group (*p* = 0.0464, Figure 4E). The results unequivocally confirmed that YKL-40 could result in progression of atherosclerotic plaque in *Ldlr* ^−/-^ mice, including lesion progression and lipid accumulation.

### YKL-40 Inhibited Apoptosis of Macrophages in Atherosclerotic Plaque of *Ldlr* ^−/-^ Mice

Macrophages and apoptotic cells in aortic root plaques of *Ldlr* ^−/-^ mice were simultaneously assayed via immunofluorescent staining (for MOMA-2) and TUNEL, respectively (Figure 4G). The percentage of TUNEL^+^ in MOMA-2^+^ cells was calculated as the apoptosis rate of macrophages in each sample. The apoptosis rate of macrophages in aortic root plaques was significantly lower in the recomb-YKL-40 group than in the IgG group (*p* = 0.0018) and anti-YKL-40 group (*p* = 0.001). However, the rate was not significantly higher in the anti-YKL-40 group than in the VC group (*p* = 0.0513, Figure 4F). The results indicated that YKL-40 exerts an inhibitory effect on apoptosis of macrophages in atherosclerotic plaques in *Ldlr* ^−/-^ mice.

### YKL-40 Inhibited Apoptosis of Macrophages *In Vitro*

**FIGURE 5:**
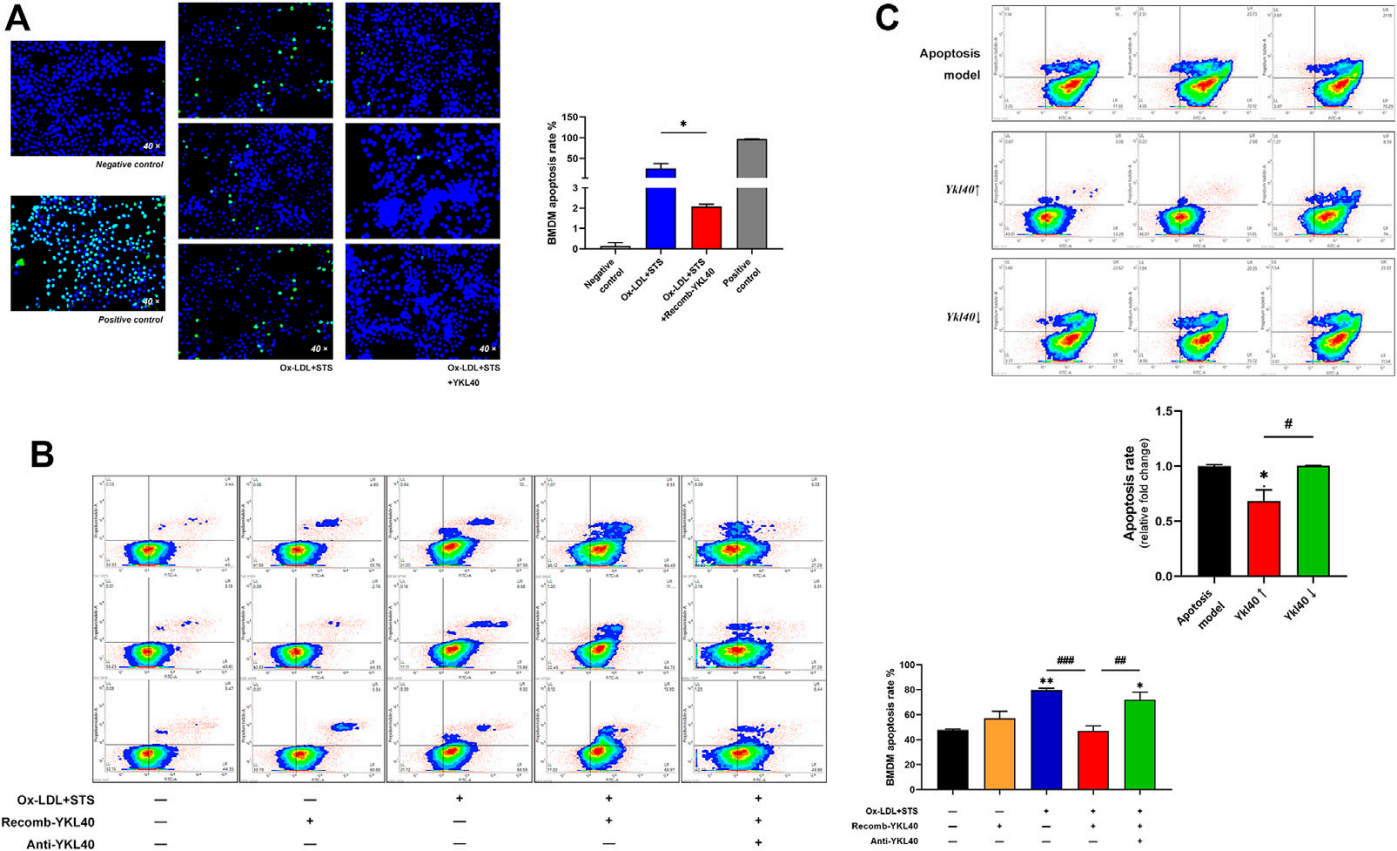
**(A)** TUNEL detection performed on BMDM. The apoptosis rate of BMDM treated by YKL-40 recombinant protein was significantly lower than that in the apoptosis model that treated by Ox-LDL accompanied by staurosporine (*p* = 0.0161). **(B)** FCM indicated the apoptosis rate of BMDM was significantly lower in the recomb-YKL-40 group than that in apoptosis model group (*p* = 0.0008), as well as that in anti-YKL-40 group (*p* = 0.0042). **(C)** FCM indicated the apoptosis rate of RAW264.7 was significantly lower in the *Ykl40*↑ group than that in apoptosis model (*p* = 0.0205) and *Ykl40*↓ group (*p* = 0.0197).

BMDM apoptosis was induced by treatment with oxidized low-density lipoprotein (OX-LDL) 100 μg/ml for 24 h accompanied by staurosporine 4 μM for 4 h. After treatment with YKL-40 recombinant protein (500 ng/ml) for 24 h on apoptosis BMDM, TUNEL detection was performed on the cells *in situ* (Figure 5A). The staining result showed that the apoptosis rate of BMDM treated by YKL-40 recombinant protein was significantly lower than that in the apoptosis model (*p* = 0.0161). Flow cytometry (FCM) was performed while another cell group, the apoptosis BMDM synchronously treated by YKL-40 recombinant protein and YKL-40 neutralizing antibody (500 μg/ml) for 24 h, was added (Figure 5B). The FCM result showed that the apoptosis rate of BMDM in the YKL-40 recombinant protein treated group was significantly lower than that in the apoptosis model group (*p* = 0.0008) and was significantly higher in the YKL-40 neutralizing antibody extra treated group than in the YKL-40 recombinant protein treated group (*p* = 0.0042). Moreover, the apoptosis rate of RAW264.7 was detected by FCM after overexpressing or silencing *Ykl40*. FCM results indicated the apoptosis rate of RAW264.7 was significantly lower in the *Ykl40* overexpressed group than that in apoptosis model group (*p* = 0.0205) and *Ykl40* knockdown group (*p* = 0.0197), but there was no significant difference between the *Ykl40* knockdown group and the model group (*p* > 0.05, Figure 5C). These results indicated that YKL-40 has an inhibitory effect on apoptosis of macrophages *in vitro*.

### YKL-40 Inhibited Macrophage Apoptosis Via Suppressing Activation of Caspase-9

**FIGURE 6:**
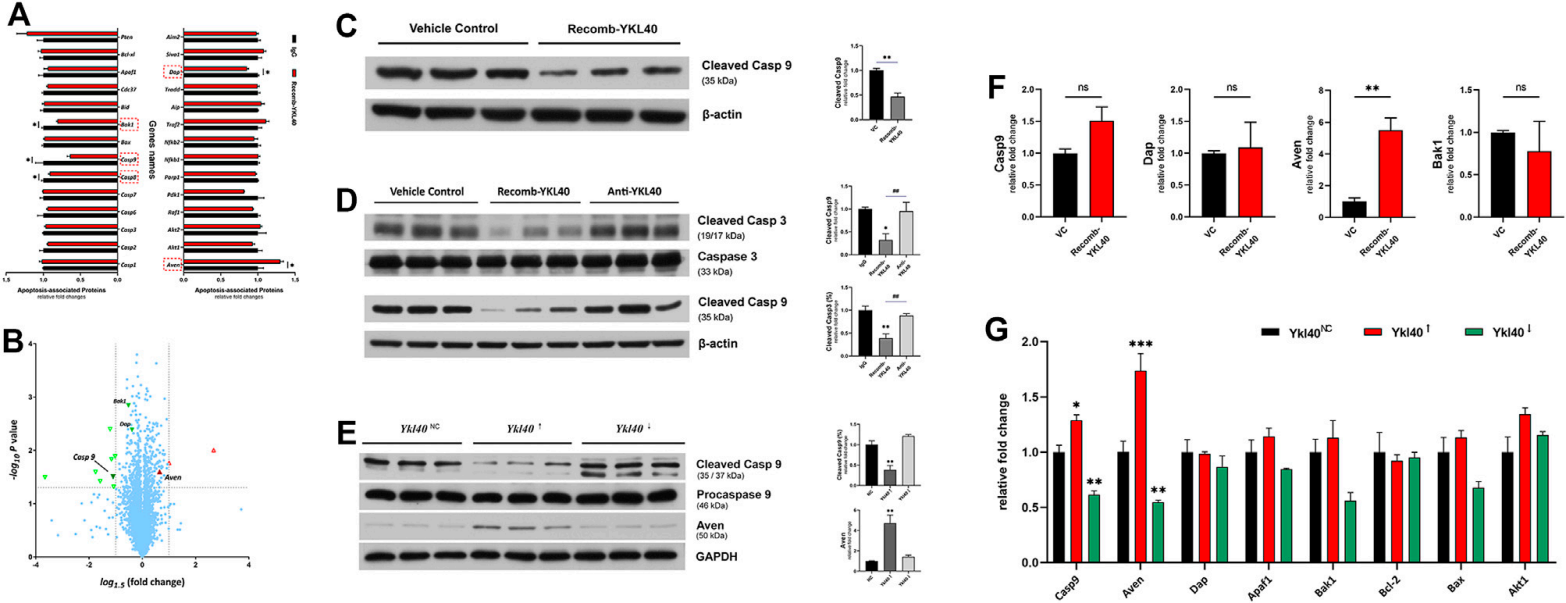
**(A,B)** iTRAQ-based proteomics analysis. **(A)** Apoptosis promotors *Casp9*, *Casp8*, *Bak1,* and *Dap* were significantly down-regulated by YKL-40, while the apoptosis inhibitor *Aven* was significantly up-regulated. **(B) *Volcano Plot***. Molecules down-regulated by YKL-40 are presented in the upper left area (green down arrow) while those up-regulated are shown in the upper right area (red up arrow), and those involved in apoptosis regulation are marked with up/down solid arrows. The most significant *fold change* (*FC*) on the expression level of these molecules was caused by *Casp9* (*FC* = 0.6411), followed by *Aven* (*FC* = 1.3006). **(C,D)** Caspase-9 expression levels detected in BMDM and aorta tissues of *Ldlr* ^−/-^ mice. YKL-40 significantly down-regulated the activation level of caspase-9. **(E)** In RAW264.7, which was upregulated by *Ykl-40*, the activation level of caspase-9 was significantly lower than that in control group (*p* = 0.0054) while the expression level of Aven was significantly higher than in controls (*p* = 0.0031). There was no significant difference in caspase-9 activation and Aven expression level between the *Ykl40* downregulated group and normal controls (*p* > 0.05). **(F)** Genetic expression relative fold changes of *Casp9*, *Dap*, *Aven* and *Bak1.* No significant difference was indicated on *Casp9* in BMDM after being treated by YKL-40 recombinant protein, but *Aven* was significantly up-regulated. **(G)** *Casp9*, *Aven* expression levels were significantly upregulated in *Ykl40* upregulated RAW264.7 (*p* = 0.0154, *p* < 0.0001) and downregulated in the *Ykl40* downregulated group (*p* = 0.0039, *p* = 0.0037).

An iTRAQ-based proteomics analysis was performed on BMDM to screen out the potential downstream target molecules of YKL-40 in regulating macrophage apoptosis. Among all the apoptosis-regulating molecules, *Casp9* (*p* = 0.0311), *Casp8* (*p* = 0.0399), *Bak-1* (*p* = 0.0014), and *Dap* (*p* = 0.0041), all of which have the effect of promoting apoptosis, were significantly down-regulated in BMDM treated by YKL-40 recombinant protein, while the apoptosis inhibitor *Aven* was significantly up-regulated (*p* = 0.0251, Figure 6A). A *Volcano Plot* revealed that the most significant *fold change* (*FC*) on expression level among these molecules was *Casp9* (*FC* = 0.6411), which was followed by *Aven* (*FC* = 1.3006, Figure 6B), *Bak1* and *Dap*.

The expression level of activated caspase-9 in BMDM were detected by western blot. The value of cleaved caspase-9/β-actin were calculated as activation level of caspase-9, which was significantly lower in the BMDM group treated with the YKL-40 recombinant protein than in IgG group (*p* = 0.0038, *p* = 0.0419, Figure 6C).

These results were verified in aortic arch tissues of *Ldlr* ^−/-^ mice. The activation levels of caspase-3 and caspase-9 were significantly lower in the recomb-YKL-40 group than in the IgG group (*p* = 0.0036, *p* = 0.0316) and that in the anti-YKL-40 group (*p* = 0.0099, *p* = 0.0420), but there was no significant difference between the anti-YKL-40 group and the IgG group (*p* > 0.05, Figure 6D).

However, there was no significant difference in the genetic expression level of *Casp9* between the recomb-YKL-40 group and IgG group in BMDM (*p* > 0.05). At the same time, the expression level of *Aven* was significantly higher in the recomb-YKL-40 group than in the IgG group (*p* = 0.0048, Figure 6F).

### YKL-40 Inhibited Macrophage Apoptosis Via Suppressing Caspase-9 Activation by Up-Regulating *Aven*

In RAW264.7 with *Ykl40* overexpressed, the activation level of caspase-9 was significantly lower than that in the control group (*p* = 0.0054), while the expression level of Aven was significantly higher than in normal controls (*p* = 0.0031, Figure 6E). However, there was no significant difference on caspase-9 activation and *Aven* expression level between *Ykl40* downregulated group and normal control (*p* > 0.05). Interestingly, although the *Aven* expression level corresponded with protein expression, the expression level of *Casp9* was significantly upregulated in *Ykl40-*upregulated Raw264.7 and downregulated in the *Ykl40* downregulated group compared with normal controls (*p* = 0.0154, *p* = 0.0039), which was quite contrary to protein presentation (Figure 6G). The results indicated the suppression effect on caspase-9 activation of YKL40 was limited to the protein modification process, while Aven could be the potential target molecule.

### Regulatory Role of Aven in YKL-40 Inhibition of Macrophage Apoptosis

**FIGURE 7:**
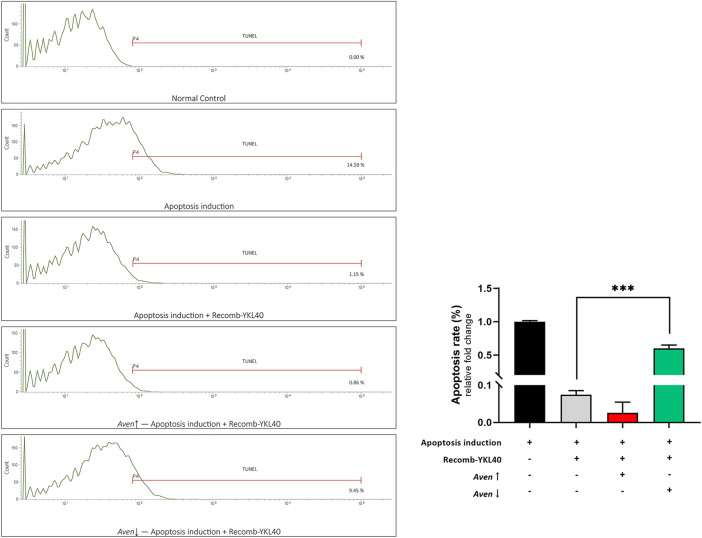
FCM analysis of the apoptosis rate of RAW264.7 after TUNEL staining. Groups are presented as normal controls, the apoptosis model that treated by Ox-LDL accompanied by staurosporine, apoptosis induced combined with YKL-40 treatment, apoptosis induced combined with YKL-40 treatment and *Aven*↑, as well as apoptosis induced combined with YKL-40 treatment and *Aven*↓. The apoptosis rate of RAW264.7 in the recomb-YKL-40 treated group was certainly significantly lower than that in the apoptosis model group (*p* < 0.001); the apoptosis rate of RAW264.7 was significantly higher in *Aven*↓ group than that in Recomb-YKL40 group (*p* < 0.001).

We modified the expression of *Aven* in RAW264.7 to confirm the effect of Aven in YKL-40 inhibition of apoptosis. FCM analysis was performed to detect the apoptosis rate after TUNEL staining. Results showed that the apoptosis rate of RAW264.7 in the recomb-YKL-40 group was certainly significantly lower than that in the apoptosis model group (*p* < 0.001). When *Aven* expression was downregulated, the apoptosis rate of RAW264.7 was significantly increased compared to that in the recomb-YKL-40 group (*p* < 0.001, Figure 7). The results suggested that the inhibitory effect of YKL-40 on macrophage apoptosis was mediated by its suppression of Aven.

## Discussion

In this study, the major findings are as follows: 1) the expression level of YKL-40 was positively correlated with the lesion size in early-stage carotid plaque; 2) the expression level of YKL-40 was negatively correlated with the macrophage apoptosis rate in early-stage carotid plaque; 3) YKL-40 treatment could suppress the apoptosis of murine macrophages and promote the progression of early-stage plaque in mice; 4) YKL-40 could inhibit the activation of the apoptosis promoter caspase-9 by upregulating the expression of apoptosis inhibitor Aven to suppress the apoptosis of macrophages.

Experimental results on the samples of early-stage carotid plaque patients indicated that the expression level of YKL-40 both in plaque tissues and in peripheral serum was positively correlated with the plaque area, suggesting that YKL-40 was significantly related to the progression of early-stage carotid plaques. Moreover, the correlation of location between YKL-40 and macrophages in plaques suggested its biological function in connection with macrophages. The results of experiments *in vivo* and *in vitro* confirmed that YKL-40 could not only promote the progression of atherosclerotic plaque but also suppress the apoptosis of macrophages in plaques*.*

Studies focused on advanced atherosclerotic lesions indicated that the elevated apoptosis rate of macrophages could enlarge the necrotic core (NC) and induce the instability of atherosclerotic plaques (Gautier et al., 2009). Many researchers are convinced that the enlarged NC has more to do with the impaired efferocytosis in later-stage atherosclerosis than macrophage apoptosis in plaques (Tabas, 2005; Schrijvers et al., 2007). However, in this study, we confirmed that in the early-stage plaque, impaired macrophage apoptosis induced by YKL-40 could certainly aggravate the atherosclerotic lesion.

Areshkov et al. thought that YKL-40 is a strong inductor of MAPK and PI3K signaling pathways, what would mediate the phosphorylation of ERK1/2 (Areshkov et al., 2012). Lee et al. have found that YKL-40 could inhibit apoptosis by inhibiting Fas (CD95) expression, activating the PKB/AKT and apoptosis inhibitor Faim3 (TOSO) (Lee et al., 2009). Unfortunately, like the most of other studies, they didn’t refer to the specific mechanism of YKL-40 regulating the apoptosis pathways. However, based on the results of the experiments on molecular mechanisms in this study, we might propose a novel apoptosis-regulated pathway of YKL-40 depending on caspases cascade and apoptosis inhibitor Aven. We analyzed the differentially expressed apoptosis-related protein molecules in BMDM through iTRAQ analysis. The results showed that the apoptosis promoters caspase-9, caspase-8, BAK, and DAP were significantly down-regulated in BMDM treated by YKL-40 while the apoptosis inhibitor Aven was significantly up-regulated. Results of the protein expression level of caspase-9, an important initial promoter of the caspase cascade, was detected in BMDM and mice tissues, consistent with the iTRAQ analysis. However, the mRNA expression level of *Casp9* detected in BMDM treated by YKL-40 was contrary to protein detection. We repeatedly confirmed these phenomena on RAW264.7 whose *Ykl40* has been up/down-regulated. The results suggested that YKL40 may not directly regulate the expression of caspase-9 but may inhibit the activation from procaspase-9 to caspase-9. Meanwhile, there was no significant difference in the mRNA expression level of *BAK* and *DAP*, while *Aven* was significantly up-regulated in BMDM treated by YKL-40.

Most apoptosis processes are facilitated by the activation of the caspase family (Thornberry and Lazebnik, 1998). Some caspases are cleaved and activated by other caspases or regulated by bridging or regulatory factors, such as Bcl-2, a prototype of the anti-apoptotic protein family, which could suppress caspase-mediated cell death (Adams and Cory, 1998; Srinivasula et al., 1998; Yang et al., 1998). Studies have identified Aven as a new apoptosis inhibitor that can bind both Bcl-X_L_, a member of Bcl-2 family, and caspase regulator Apaf-1, suppressing apoptosis induced by Apaf-1 plus caspase-9 (Chau et al., 2000). In this study, we confirmed by silencing or over-expressing *Ykl40* in RAW264.7 that Aven was significantly regulated by YKL40 in both protein and mRNA expression levels. Moreover, flow cytometry on RAW264.7 confirmed that the suppressive effect of YKL-40 on macrophage apoptosis was significantly impaired when *Aven* was down-regulated.

Based on the above results, we suggest that YKL-40 can up-regulate the expression of Aven which could suppress the activation of caspase-9 by interfering with APAF-1 binding to it, then inhibit the activation of the caspase cascade and finally inhibit the apoptosis of macrophages. Moreover, in our previous study, we have found YKL-40 also exert an inhibiting effect on macrophage efferocytosis, a key part of PrCR. Which suggest YKL-40 might play an important role in impairing the entire process of PrCR in atherosclerotic lesion. However, it is ambiguous that YKL-40 promotes atherosclerosis mainly on account of its inhibiting effect on apoptosis or efferocytosis, and the specific mechanism of YKL-40 up-regulating Aven expression remains elusive. Which are worthy of further research and discussion. In addition, administration process that we performed in this study might provide more solid evidence on the global effect of YKL-40 affecting the plaque progression than the local effect *in Vivo*. However, it is our conjecture based on existing positive results that the YKL-40 might impair the PrCR to impact plaque macrophages and then aggravate atherosclerosis. Locally intervening experiments would be arranged to further confirm the local effect of YKL-40 in the future study.

**FIGURE 8:**
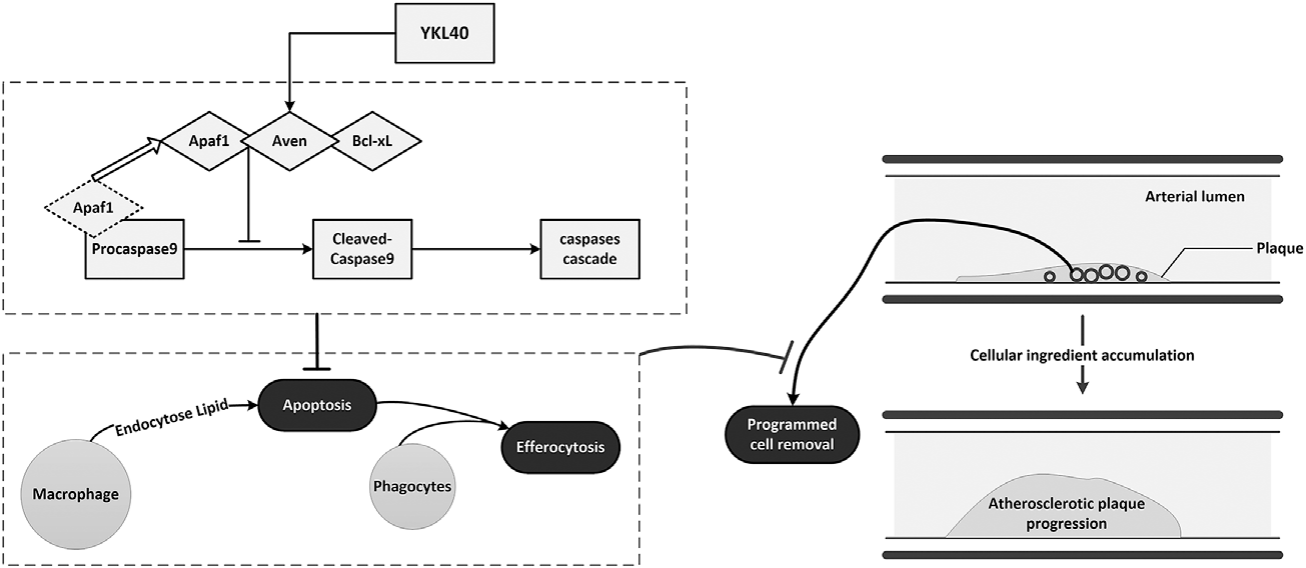
Schematic diagram of the mechanism of YKL-40 aggravates early-stage atherosclerosis by inhibiting macrophage apoptosis in an Aven-dependent way.

In conclusion, YKL-40 might suppress the apoptosis of macrophages by inhibiting the activation of apoptosis promoter caspase-9 via up-regulating the expression of the apoptosis inhibitor Aven. Inhibited apoptosis of macrophages results in impaired PrCR in plaque. The accumulation of macrophages that should have been apoptotic and removed by efferocytosis could enlarge the plaques and aggravate early-stage atherosclerosis (Figure 8).

## Data Availability

The datasets presented in this study can be found in online repositories. The names of the repository/repositories and accession number(s) can be found below: ProteomeXchange, PXD028305.
