# Supplementary material for: YKL-40 Aggravates Early-Stage Atherosclerosis by Inhibiting Macrophage Apoptosis in an Aven-dependent Way
Source: Front Cell Dev Biol. 2021 Dec 7;9:752773. doi: 10.3389/fcell.2021.752773 (PMC8688858; doi:10.3389/fcell.2021.752773)
Supplement: Supplementary file 1 [file Table1.PDF]

Supplementary Table 1. Baseline Information

	Small (n=10)	Large (n=11)	*P*
**Age (years)**	65.70±1.438	69.64±2.671	0.2228
**Gender (M/F)**	9:1	7:4	0.3108
**Weight (kg)**	71.95±3.711	68.45±3.220	0.1418
**Medical History (%)**			
**smoking**	50.00	54.55	>0.9999
**hypertension**	54.55	45.45	0.6699
**diabetes**	30.00	63.64	0.1984
**CHD**	10.00	0	0.4762
**amaurosis fugax**	20.00	45.45	0.3615
**TIA**	20.00	18.18	>0.9999
**stroke**	30.00	27.27	>0.9999
**Drug use (%)**			
**aspirin**	70.00	54.55	0.6594
**clopidogrel**	0	9.09	>0.9999
**statin**	60.00	45.45	0.6699
**ACEI**	40.00	9.09	0.1486
**β-blocker**	10.00	18.18	>0.9999
**CCB**	30.00	0.00	0.0902
**Laboratory examination**			
**SCr (μmol/L)**	85.80±7.31	79.18±4.03	0.4264
**TCHO (mmol/L)**	3.98±0.16	3.74±0.31	0.5105
**TG (mmol/L)**	1.29±0.09	1.14±0.16	0.4731
**LDL (mmol/L)**	2.36±0.10	2.02±0.15	0.0805
**HDL (mmol/L)**	1.03±0.05	1.12±0.05	0.2075
**HCY (μmol/L)**	13.70±1.14	13.22±0.82	0.7329
**Plaque CSA (mm^2)**	3.29±0.48	9.08±1.86	**0.0096**
**Total intima CSA (mm^2)**	7.84±0.83	12.51±1.76	**0.0317**
**Plaque area (%)**	40.35±2.79	67.98±4.33	**<0.0001**

TIA, transient ischemic attacks; CHD, coronary heart disease; ACEI, angiotensin-converting enzyme inhibitors; CCB, calcium channel blocker; SCr, serum creatinine; TCHO, total cholesterol; TG, triglyceride; LDL, low density lipoprotein; HDL, high density lipoprotein; HCY, homocysteine; CSA, cross section area.
