# Supplementary material for: YKL-40 Aggravates Early-Stage Atherosclerosis by Inhibiting Macrophage Apoptosis in an Aven-dependent Way
Source: Front Cell Dev Biol. 2021 Dec 7;9:752773. doi: 10.3389/fcell.2021.752773 (PMC8688858; doi:10.3389/fcell.2021.752773)
Supplement: Supplementary file 2 [file Image1.PDF]

Supplementary Figure

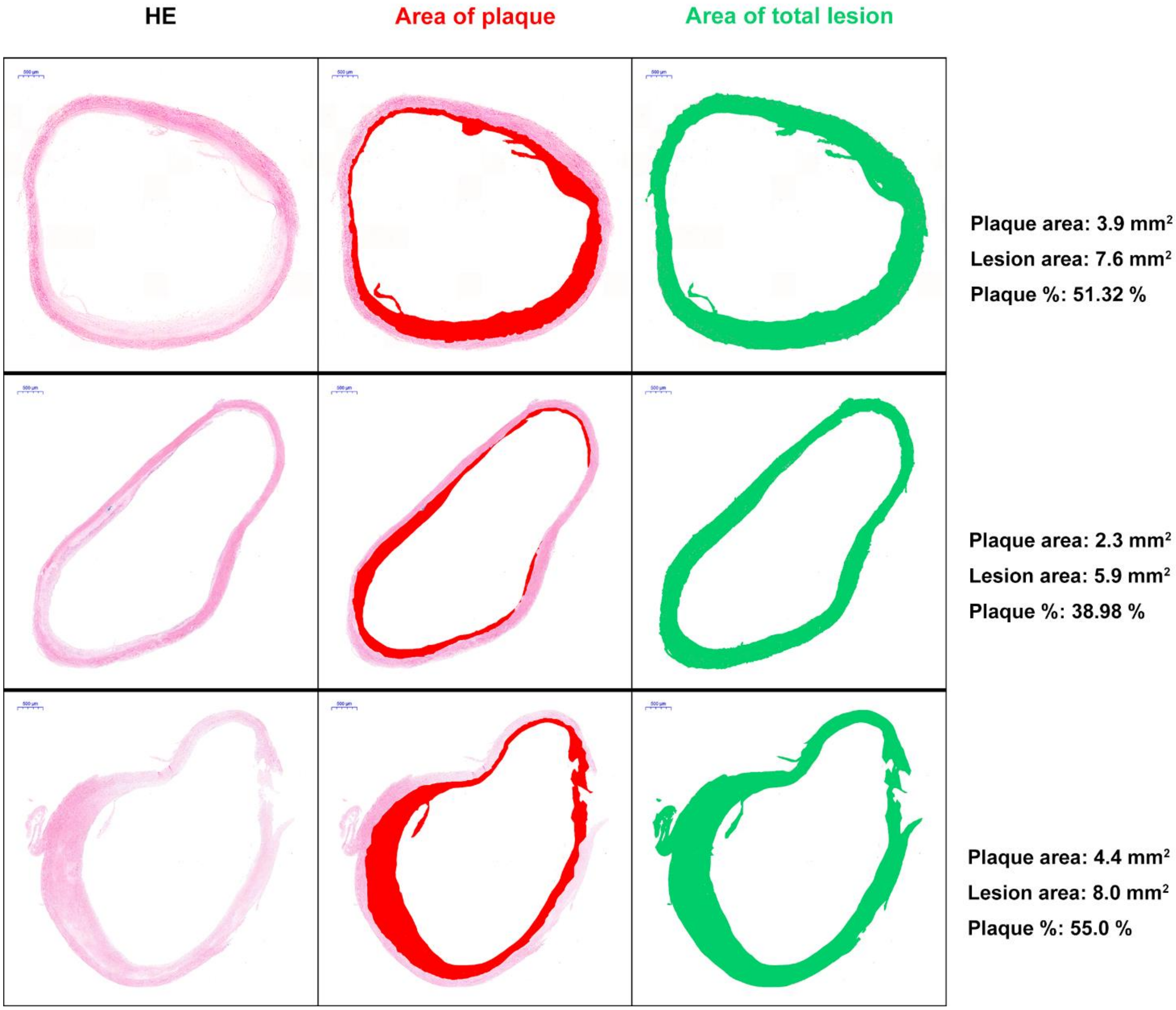

Supplementary Figure 1. The cross-sectional area of plaque (red area) and total lesion (green area) of carotid plaque samples were respectively calculated according to H&E staining outcomes. The percentage of plaque area to intima area was counted as the relative plaque area (%) of each sample.
